# The Management of Infancy, Physiological and Moral

**Published:** 1861-01

**Authors:** 


ART. II.—*The Management of Infancy, Physiological and Moral. Intended chiefly for the Use of Parents.* By ANDREW COMBE, M.D., Fellow of the Royal College of Physicians in Edinburgh, and one of the Physicians in Ordinary to the Queen. Ninth Edition. Revised and Edited by SIR JAMES CLARK, Bart., M.D., F.R.S., Physician in Ordinary to the Queen and His Royal Highness the Prince Consort. Edinburgh: Maclachlan & Stewart, 1859. pp. 302. 12mo.

FEW writers have been so successful in disseminating a knowledge of practical or applied hygiene as Dr. Combe has been in the successive works which he issued on this fruitful theme; beginning with the one entitled "*The Principles of Physiology applied to the Preservation of Health, and to the Improvement of Physical and Mental Education,*"† and ending with that now before us, which made its first appearance in 1840. In the intermediate period, in the year 1836, he sent out "*The Physiology of Digestion, considered with reference to the Principles of Dietetics.*" He also edited the work of Dr. Beaumont on the *Gastric Juice.*‡ The first work written by Dr. Combe was entitled "*Observa-*

---

* Hist. de l'Anat. et de la Chirurg., tome i. p. 399. † First published in 1834.

‡ Experiments and Observations on the Gastric Juice and Physiology of Digestion.

*tions on Mental Derangement*," based on the principles of physiology—1831. It was favorably spoken of by Dr. Abercrombie. His extreme conscientiousness prevented him from bringing out a second edition, as his other avocations and infirm health would not allow of his giving that attention to the treatment of insanity and the progressively increasing knowledge of the entire subject, which its importance demanded.

The first edition of the Management of Infancy, etc., was dedicated to Sir James Clark, the Queen's physician, and now, in a spirit of the kindest regard and admiration of the author, Sir James, has "revised and edited" this, the ninth and last edition. In a well written and appropriate "Introduction," the editor, after assigning the motives which influenced him on the occasion, says: "The work has been to me a labor of love, and it has not been a heavy one, as the last edition, which the author himself corrected, was left so perfect that little remained for an editor to do." We cannot commend the freedom taken by Sir James in his having altered the order of some of the chapters, even though it has been done "with the view of bringing the subjects treated of more consecutively before the reader;" nor his having ventured to omit some portions, chiefly in the earlier chapters, as less necessary now than during the author's life. The most exacting reader would, it seems to us, have been quite content to receive, without alteration or curtailment by another hand, the matured opinions, as well as the language in which they were couched, of the author himself, after the opportunities which he enjoyed for accomplishing these objects in six successive editions of his work. By no critic can Dr. Combe be accused of redundancy and needless repetition, nor of obscurity in the general management of his subject, or want of method in its details. There is a decided inconvenience in the transposition of chapters and omissions of parts of the text by an editor, in its producing embarrassment and confusion when referring to and quoting from the work. There will be a want of correspondence between the original by the author himself, and the edition moulded to suit the taste of his friend and commentator. Thus, for instance, had a writer quoted a passage from the first chapter of the sixth edition of Dr. Combe's work, treating of the *mortality in infancy*, etc., the reader of a copy of the present edition, who had not seen a previous one, would accuse him of inaccuracy and wrong citation, and would correct him by saying that this is the subject of chapter third. Still more serious would be the confusion produced by one writer quoting a passage which another could not find in the volume; the former having made use of the author's edition, the latter having access only to the editor's, and *e cenverso*, the language of the latter may be erroneously cited as that of the former. We need not press the argument further, and would scarcely have thought it worth while to do more than to enter a brief protest in the present instance, were we not apprehensive that an example thus set by Sir James Clark, with the most amiable motives, might be followed by others on a larger scale, and under equivocal circumstances. Some of the additions made by the editor of the present work are incorporated in the text: they would more appropriately, for the reasons which we have assigned, find a place in the form of notes, or in the appendix actually made, and which consists of useful matter germain to the work.

We pass with pleasure from this brief criticism to more congenial themes—the decided and distinctive merits of the labors of Dr. Combe in the present volume, and the hearty and discriminative appreciation of them by Sir James Clark, in his "Introduction." Addressing himself more particularly to the different parties who would be greatly benefited by a careful perusal of the work, the editor says:—

"My great aim has been to carry out what I know was the object of the author, namely, to make the work useful to young medical practitioners and teachers of youth, as well as parents, for whose guidance the work was mainly and originally designed. To my younger medical brethren, entering on the anxious and responsible duties of their profession, I earnestly recommend a careful study of the book—I know none on the subject of infant hygiene in which is to be found so much valuable practical information to guide young medical practitioners in the management of infants and young children. I venture to give the same advice, and with equal earnestness, to teachers, both male and female, and more especially to the teachers of infant schools."

The editor, in continuation, insists on the importance of a thorough understanding of the principles so lucidly explained by Dr. Combe, respecting the progressive development of the mental faculties, and the application of such knowledge by those who have the management of infant schools. He considers the same advice strictly applicable to governesses. "How important is it then that this class of teachers should possess such a knowledge of the physical and mental constitution of the young beings committed to their charge, as shall enable them to cultivate and train the moral feelings as well as to instruct the mind!" And, a few lines farther, he makes the following emphatic declaration: "In my opinion *no teachers of any class should be considered competent for their duties till they have given proof of possessing a general knowledge of the structure and functions of the human body and of the laws of health.*"

"Within the last few years," as we learn from Sir James Clark, "physiology, in its application to health, has been taught with considerable success in several seminaries, more especially in the Birkbeck schools in London, under the enlightened direction, and by the liberal support of Mr. William Ellis, and it has been invariably found, even at a very early age, to excite in a remarkable degree the interest and attention of the pupils. It is to be hoped, therefore, that the time is not far distant when physiology will form an essential part of the course of instruction in every school. It is in vain to expect that education can be rightly and successfully conducted until the *educators* themselves are instructed in the nature of the physical and mental constitution of those whom they undertake to train and instruct."

In a note the reader is referred to the Appendix E, for the opinion of sixty-five of the most eminent physicians and surgeons in London, including the professors of anatomy and physiology, strongly expressive of their belief of the benefits to be derived from making the elements of human physiology, or a general knowledge of the laws of health, a part of the education of youth. They "are convinced that such instruction may be rendered most interesting to the young, and may be communicated to them with the utmost facility and propriety in the ordinary schools, by properly instructed schoolmasters." Dr. Combe himself makes an earnest appeal, which the editor has transferred from the body of the work to his Introduction, urging the necessity of an acquaintance with physiology and the constitution of the infant frame being made an essential part of *female education.* We quote the concluding paragraph:—

"It may indeed be alleged, that mothers require no knowledge of the laws of the infant constitution, or of the principles of infant management, because *medical aid* is always at hand to correct their errors. According to the present habits of society, however, professional men are rarely consulted till the evil is done, and the health broken; but even if they were, intelligence and information are needed in the mother, to enable her to fulfill their instructions in a rational and beneficial spirit. On every account, therefore, it is urgently necessary that female education should be such as to fit both mind and body for the duties as well as for the embellishments of life, for the substantial happiness of the domestic circle, at least as much as for the light and fleeting hours of fashionable amusements; and that, while every effort is made to refine and elevate the mind, the solid substratum of useful knowledge should not be neglected."

Reference is made by the editor to "The Ladies' National Association for the Diffusion of Sanitary Knowledge," the principal object of which is the preservation of the health of children. "It is to women that we must look first and last," says Miss Nightingale, "for the application of sanitary knowledge, as far as household hygiene is concerned." Sir James Clark offers a very pertinent suggestion "for the consideration of those ladies who have gone through a systematic course of medical education, with a view to qualify themselves as medical practitioners, as to whether devoting their time to the instruction of their own sex in the laws of health would not form an equally useful and a more appropriate profession than that of a physician or surgeon?" The suggestion is well timed, and if adopted, would place the female teachers of their own sex on a higher ground and in a more advantageous position than can be occupied by any of their number as practitioners of medicine.

Hygiene, as justly remarked by the editor, has not received the attention which it merits as a branch of medical education. "The University of London is," he believes, "the only one in the country in which hygiene finds a place among the prescribed subjects of examination for degrees in medicine—a regulation mainly owing to Dr. Combe's earnest recommendation."

"In the Army Medical School just instituted, *hygiene* will form the most important branch of the young medical officer's instruction. For originating this school we have to thank Miss Nightingale, who, had her long and persevering efforts effected no other improvement in the army, would have conferred, by this alone, an inestimable boon upon the British soldier, and merited the gratitude of the country for the improved sanitary condition of the army, and the saving of human life, which will be the result."

Ought there not to be in all the military posts of the United States, not only riding schools for the cavalry soldier, but also gymnasia for soldiers of all arms, in which they might be trained to various exercises and feats of strength and agility, with the immediate effects of preserving health and furnishing amusement, and the more remote and contingent, but still very important ones of imparting to them more powers of endurance and greater efficiency in a campaign, and especially when detached to reconnoitre, or as skirmishers, and in a retreat?

We cannot refrain from one more quotation from the "Introduction;" the sentiments conveyed in it are coincident with those which we have repeatedly expressed on the subject:—

"When the public is made fully aware of the extent to which health may be preserved and disease averted and mitigated by a knowledge of physiology, the practice of medicine will assume a very different aspect from that which it presents in its present unsettled state. The physician will then be more frequently consulted on the means of preserving and improving health than for the treatment of disease, as is almost solely the case at present."

We shall not offer any apology for introducing those preliminary matters before entering on a formal notice of the contents of the work of Dr. Combe. If we have kept our readers on the threshold, it was that they might enjoy the company and discourse of the editor, Sir James Clark, the friend of the author, while touching on kindred themes, and enhancing the relish for that which is to come. It will be remembered, also, that we have not to deal with a new work. "The Management of Infancy" has gone through successive editions in the United States. The first American followed, after a few months in the same year, (1840,) the first English edition. It received numerous notes and a supplementary chapter from Dr. Bell, who undertook this task in order to give Dr. Combe the pecuniary advantage which ensued from the publishers being able to take out a copyright on this side of the water.* The volume was first published from stereotype plates, by Messrs. Carey & Hart, of Philadelphia. These plates are now in the possession of Messrs. Fowler & Wells, of New York, who have published, at different intervals, several editions.

The themes severally discussed by Dr. Combe in this volume are of the highest importance to the well-being and usefulness of mankind. Though turning mainly on the physical and moral training of infancy, they have also an obvious bearing on adult life; much of the happiness of which will depend on the manner in which the first mentioned period has been passed. Following the order of succession of the different chapters as they present themselves in the present edition, we meet, in chapter first, with the views of the author on the "Influence of the Constitution of Parents on the Health of their Children." The subject does not come within the domain of love and sentiment, and is seldom thought of in forming matrimonial engagements, although it is not long before the discovery is made, that young romance cannot remain in its dream-land when the objects of first admiration become the victims of bodily infirmities and decay, with their too frequent concomitants of caprice, peevishness, and vagaries of fancy in which ill-timed suspicion performs an important part. Dr. Combe contrasts "some families apparently surrounded by every external advantage, yet in which it is found difficult to rear the children to maturity," with other families seemingly much less fortunate in their outward circumstances, but in which one child grows up after another, as if no such thing as disease existed, or as if the ordinary disorders of infancy were but mysterious processes for the further development of the organism." The difference in these cases is dependent on some inherent difference of constitution derived from one or both parents, which is commonly spoken of under the title of hereditary influence.

---

* That this voluntary service was appreciated by Dr. Combe in the spirit in which it was offered, is evident from the following lines in the Preface to the sixth and the last edition which he ever supervised: they carry with them the more interest as they were written within two months of his death, which took place August 9th, 1847:—

"Here I cannot resist the opportunity of expressing my grateful acknowledgments to Dr. John Bell, of Philadelphia, for the time and trouble which, amid many pressing avocations, he so kindly and disinterestedly bestowed, not only in superintending the republication of this work in the United States, but in enriching it with many valuable notes, for the purpose of adapting it more completely to the domestic habits and wants of the Transatlantic public."

The conditions in the parents which affect most powerfully the health of the child are thus stated :—

"*Natural infirmities of constitution, bodily and mental, derived from their parents.*

"*Premature marriages, especially of delicate persons, and of those strongly predisposed to hereditary disease.*

"*Marriages between persons too nearly allied by blood, particularly when either of them is descended from an unhealthy race.*

"*Marriages contracted too late in life.*

"*Great disproportion in age between the parents.*

"*The state of the parents at the time of procreation.*

"*The state of health, and conduct, of the mother during pregnancy.*"

On the subject of "hereditary predisposition," the author adverts to the influence of descent, manifested even in the vegetable as it is in the animal world :—

"Quite as much importance is attached to the quality of the seed as to a good soil and good cultivation ; and in like manner in rearing the lower animals, especially the horse, purity of race is prized above all other conditions; and so entire is the dependence placed upon the hereditary transmission of qualities, that the genealogy of the race-horse, of the hunter, and even of the farm-horse, is regarded as a sure criterion of the properties which may be expected in their progeny. In the dog, the sheep, and the different varieties of cattle, we calculate with certainty on the reappearance of the qualities of the parents in their young."

Man himself constitutes no exception to the general law. "In relation to this subject the great Haller mentions a very remarkable instance of two noble ladies who were married on account of their wealth, although they were nearly idiots; and from whom the mental defect extended for a century into several families, so that some of all their descendents continued idiots into the fourth, and even to the fifth generation." Examples without number might be cited to the same purport. The results of descent work both ways. "The very same law by which the liability to gout, insanity, imbecility, scrofula, and constitution is transmitted from generation to generation, enables us to reckon with equal certainty on the transmission of health and vigor wherever these have been the hereditary features of the race." The practical inference from these laws is obvious, the penalty for neglecting it is equally clear and certain in its exaction.

The evils from the "marriage of persons too nearly allied in consanguinity" have been exhibited on a large scale "in the deteriorated off spring of some of the royal and aristocratic families of Europe, in which frequent intermarriages have taken place without any regard being paid to the morbid predisposition on either side. Prejudices of race and religion, as in the instance of the Jews, have led to similar intermarriages, and under disastrous results. "The Jews in England," says Dr. Elliotson, "have the same bad practice of marrying first cousins; and I never saw so many instances of squinting, stammering, peculiarity of manners, imbecility, or insanity, in all their various degrees, intense nervousness, etc."

It is not long since that the public papers contained an account of the marriage ceremonies, the bridal trousseau, and magnificent feast, on the occasion of the union of two cousins, a daughter of the English Rothschild, and a son of the German one. How much it is to be regretted that the two fathers who have such an intimate knowledge and keen perception of the workings of the stock exchange, should not have acquired some elementary information on the results of the kind of dealing in family stock, in which they allowed themselves to be engaged on this occasion, with every probability of a terrible depreciation of marriage investment at an early day. The editor refers, in the Appendix, to the results of marriages of consanguinity in the United States, as stated by Dr. Bemis, of Kentucky, in the first volume of the *North American Medico-Chirurgical Review*, and by Dr. Devay, "Hygiene des Familles," in the *American Journal of Insanity*, 1859.

Taking it "as the general rule that women do not attain their full development before from twenty to twenty-three years of age, and men from twenty-five to thirty," a statement nearly applicable to this country as well as to Great Britain, we can easily conceive the detrimental effects of delicate girls marrying at seventeen, and sometimes even at fifteen, "before the mental or physical powers are fully developed, and at the manifest risk of entailing infirm health on themselves and their offspring, and thus throwing away the best chance of their own permanent happiness." "Late marriages are scarcely less unfavorable to the health of the offspring than those contracted in very early life."

The state of the parents at the time of procreation, the most frequent and the most injurious to the offspring, is *habitual indigestion* and its consequences, *impaired nutrition.* The objection often urged against the hereditary transmission of certain qualities of mind, that "clever men generally have stupid children," is met by the following, among other remarks, by Dr. Combe: "Not to dwell on the circumstance that men of genius frequently marry late, it is notorious that nothing can be farther removed from the standard of nature than their state of health and general mode of life. Are they not, as a class, enthusiastic, excitable, irregular in their habits, the sport of every passing emotion, and almost without exception, victims to indigestion and often to melancholy?"

"The Influence of the Mode of Living during Pregnancy on the Health of the Child," is the subject of chapter second. In it the author treats of mental emotions, longings, diet, dress, exercise, bathing. To contribute to the health, comfort, and cheerfulness of the mother during pregnancy, is the paramount duty of all who are brought in relation with her during this time, and at none other is a rational observance of the laws of health more necessary. Healthful, invigorating occupation, exercise of mind and body, the use of plain, nutritive food and air, and an avoidance of stimulants, ought to be especially enjoined. The pregnant female should "be on her guard against giving way to caprice of temper, to the temptations of indolence, to endless novel-reading, or to any form of social dissipation." If to these we add great cleanliness, moderate diet, pure air, early hours, clothing suitable to the season, and healthy activity of the skin, we shall have specified all the conditions to be complied with in this most interesting, if not the most trying period of woman's matrimonial life.

The "Causes of Infant Mortality" are next examined with care, in the third chapter of the volume, and many of them shown to be removable, and to be increased by parental ignorance. We cannot follow the author in his statistical proofs of the several positions advanced, but copy his summing up as follows:—

"From these incontrovertible facts, corroborated by returns derived from other countries, [in addition to England,] the reader may conceive how many *elements* of destruction must still be in activity, even in those parts where science has made the greatest advances, and where the treatment of the young is considered the most rational, when *one in* every seven infants ushered into the world perishes within the first year, and one in every five within the first two years of existence!" The enormous mortality in children is under the first five years of their age. A strong, it may be called startling fact, confirmatory of there being preventible means against the great mortality among children of a tender age, is quoted by Dr. Combe. About a century ago, the workhouses of London presented the astounding result of *twenty-three deaths in every twenty-four* infants under the age of one year. Under an improved system of management, in consequence of a Parliamentary inquiry, the proportion of deaths was speedily reduced from 2600 to 450 a year! "There then was an annual loss, in a single institution, of 2150 lives, not attributable to any unalterable decree of Providence, as some are disposed to contend, as an excuse for their own negligence, but to the ignorance, indifference, or cruelty of man!" Another instructive example of the extent to which infant mortality may be diminished by rational treatment, is furnished by Dr. Joseph Clarke, from the Register of the Lying-in Hospital of Dublin. Struck with the extraordinary mortality, which at the conclusion of 1782 was nearly every sixth child, and which seemed to him to be owing to the impurity of the air, he procured better ventilation, and the proportion of deaths was speedily reduced to one in nineteen and a half. Mr. Simon, the medical officer of the Privy Council, quoted by Dr. Combe, very justly remarks, that the "*death-rates* of young children are among the most important studies in sanitary science, not only on their own account, but as affording a very sensitive test of the sanitary circumstances of the district." "A high local mortality of children almost necessarily denotes a high local prevalence of those causes which determine a degeneration of the race."

In chapter fourth, the author shows, with his customary force and clearness, that infant health is not accidental, but dependent upon fixed laws. For a better understanding of the subject by the reader, an intelligible account is given of the infant constitution, and of the chief conditions by which infant health is influenced, so that it may be practically useful in the hands of any parent of ordinary capacity. The author inculcates the fact, "that disease and untimely deaths are the result, not of chance or of any abstract necessity, but simply of the infringement of the *conditions* on which God has decided the healthy action of the organs of the body to depend, and which have, therefore, been appropriately named the *organic laws*." "The grand aim, therefore, in attempting to improve the treatment of infancy, ought to be the discovery and fulfillment of the conditions on which the healthy action of the principal organs and functions depends."

Carrying out the principles laid down, the author proceeds, in chapter fifth, to speak of the constitution of the infant at birth. To the general reader, a knowledge of this kind is highly necessary: to our professional brethren, it carries with it no novelty in the fact, although it is made more instructive from the manner it is presented. The still popular idea that infants have a great power of resisting external cold is combated and its fallacy exposed. There is hardly a more pernicious error in the management of infancy than this one, and its absurd sequences of hardening, as it is called, the little being by careless and cruel exposure of parts of its body to cold, which are, in fact, the most sensitive, and require the argest measure of protection against this debilitating agent. The proper balance between the organs of *nutrition* and those of excretion—between the supply and the waste—is pointed out as an essential condition of health, not only in infancy, but at every period of life. The organic and the animal functions, and their relative influence on the organism, are described.

Chapter sixth teaches "The Management of the Infant immediately after Birth." The details under this head we must suppose are familiar to the practicing physician, and, therefore, need not be repeated here. In one important particular, professional opinion, or, at least, passive consent, favors the absurdities of fashion. We refer to the matter of dress of infants, whose comfort and health would seem to be the last thing thought of by mothers who usually have the control on these occasions.

In the chapter on the "Food of the Infant," much wholesome advice is offered on the nature and action of the mother's milk, the intervals between suckling, and the regular periods for this process, which ought to be observed from the earliest infancy, and attention to which will prevent the mother from letting the infant go to sleep on her bosom, and tug at short intervals at the nipple. The mode of life of the mother when nursing, and the food of infants on the appearance of the first teeth before weaning, also the manner and best time for weaning, and caution against the abuse of medicine, are topics brought up and discussed in due succession.

"Cleanliness, Exercise, and Sleep in Infancy," are the topics of chapter eighth. "In infancy, cleanliness is of the first importance to health," and the requirements in this respect are to be carried out by immersion of the entire body in a warm bath suitably arranged for the purpose, as far preferable to the imperfect mode of washing in a tub, or, worse still, on the knees of the mother or nurse. "The best time for washing it is in the morning as soon as it is taken out of bed; and for bathing, the evening, after its last meal, and before being put to sleep." The younger the child, the more necessary is it to adhere to a particular standard of temperature of the water: this should be 96° F., and gradually reduced as the child grows older, to that of a tepid bath. Fresh air is an important, but much neglected supplement and auxiliary to the bath, especially when this is taken at a reduced or cold temperature. The enjoyment by the child of the air bath of Franklin is recommended. As regards exercise, "the child can scarcely be too much in the open air, if the morning and evening chills and dews be avoided," it being understood that the period should be cautiously and gradually extended, from fifteen to twenty minutes at first, to an hour or two, and at last to several hours a day in proportion as it can be borne. A *sitting posture* for the infant should be carefully avoided by the nurse, for at least the first four or five months after its birth. The nurse should change from time to time the arm on which the child rests. "The common custom of dandling, swinging, and jolting of very young infants is highly improper." On the subject of sleep, notice is taken of the inconceivably great mischief done by many mothers and nurses who, in order to quiet a restless child, are in the habit of resorting immediately to laudanum, sedative drops, spirits, and other means of an analogous nature. "Flowers and strong smelling perfumes should be excluded from the sleeping apartments of infants, as they act injuriously on their nervous system." The prohibition ought to be extended to the sleeping rooms of persons of all ages.

"On the Choice and Regimen of a Nurse," the subject of chapter nine, we find excellent advice. "A tranquil and even temper are particularly desirable in a nurse, and care should be taken to inquire into this point." In the choice of a nurse a well-informed physician should always be consulted. "Artificia nursing" ought never to be had recourse to, unless the mother is unable to suckle the child, or a suitable nurse cannot be procured. Reference is made in this chapter (ten) to the Appendix, in which will be found a full account of the milk of different animals compared with that of women.

Much wise remark and advice will be found in chapter eleven, on "The Nursery and Conditions required in it." Beginning at the foundation, the author treats of the *site of the house*, the *nature of the soil* on which it stands, the *position of the nursery, the ventilation of the nursery.* Additional illustrations are given in proof that the higher mortality in cities is owing to the insalubrity of the atmosphere, and one of the great supports of this morbid condition is the want of ventilation. "However suitable in size and situation the nursery may be, adequate *ventilation*—that is, a *frequent*, and still better, a continuous *renewal* of the air contained in it—is indispensable to health." This advice is not meant, however, to imply exposure of the young inmates to cold draughts or sudden cooling of the air of the room.

With equal wisdom does the author discourse, in subsequent chapters, on the "Management of the Infant during Teething," and of "The Child from Weaning to the end of the Second Year." For the elucidation of the higher questions of "Mental Constitution and Principles of Training in Infancy," "Moral Education in Infancy and Childhood," and further remarks on the "Moral Management of Infancy and Childhood," we must look to the concluding chapters bearing these headings. It is here that the true philosopher shows himself, as a careful delineator of the actual compound condition of the young being, and a drawer of logical sequences for the intellectual and moral guidance, or the proper education of childhood. Truly are we told that, as "whatever tends to modify the corporeal or mental constitution of man tends to have a permanent influence on him for good or for evil, it follows that although education, technically so called, is generally delayed till the age of five or six years, real education, or the influence of surrounding circumstances, begins at birth, and often lays a durable foundation for the future bodily and mental character, even before the dawn of distinct consciousness."

We shall close this notice of Dr. Combe's work referring to the probable causes of his attention having been early directed to the subject of the management of infancy. They were of a personal nature, and grew out of the circumstances under which his own childhood was passed. His father,

George Combe, as we learn from the Life and Correspondence of the Doctor, written by his brother, George Combe, was a brewer in Edinburgh. The spot on which the family resided was called "Livingston's Yards;" it was low, damp, and in winter shaded from the sun; and the dwelling-house was insufficient to afford comfortable accommodation to the large family which inhabited it. Andrew was the fifteenth child and seventh son, and, in common with all the members of this numerous family, suffered largely from the vitiated air generated in close and imperfectly ventilated rooms, especially during the night. The same want of aeration was exhibited in the clothes and bedding; and if to the neglect, in these ways, we add that of general ablution or bathing, the more surprising, where warm water was constantly at hand, we shall find an explanation of the appearance of disease and impaired constitution in the children. Such is the description, by his brother George Combe, of the want of sanitary practice, the result of ignorance of the subject, at "Livingston's Yards;" and such is the conclusion drawn by Dr. Combe, in a letter to his brother George. The following paragraph in the same letter conveys a reproof which we fear is not undeserved by a goodly number of the medical profession at the present day:—

"Our parents erred from sheer ignorance, but what are we to think of the mechanical and tradesmanlike view of a medical man who could see all these causes of disease subsisting and producing their results for year after year, without its ever occurring to him that it was part of his solemn duty to warn his employers, and try to remedy the evil? All parties were anxious to cure the disease, but no one sought to remove the cause; and yet so entirely were these causes within the control of reason and knowledge, that my conviction has long been complete, that if we had been properly treated from infancy, we should, even with the constitutions we possessed at birth, have survived in health and active usefulness to a good old age, unless cut off by some acute disease. In my individual case, I can trace with ease the causes which have combined to impair my stamina and cut short my life; and almost every one of them could have been avoided with facility, had either our parents possessed the knowledge which I have endeavored, by my writings, to convey to other parents, or their medical advisers had a proper sense of their own duties, or of the responsibilities attached to them. Under all disadvantages, I have shown a tenacity of life which leaves a very strong presumption that, under good management, I might have gone through an active life of seventy years."*

Dr. Combe died August, 1847, a little before the completion of his fiftieth year. He had been the subject of frequent attacks of pulmonary disease, which on several occasions brought him to the brink of the grave. "Such, indeed, was his condition," as remarked by Sir James Clark,

---

* Life and Correspondence, pp. 34, 35, Am. edit.

"that it might be truly said of him that the greater part of his life was one long disease." He spent winters in the south of France and in Italy, and, at a later period, two seasons in Madeira. His remarks on the climate of these places, and of their fitness as a residence for invalids, in letters to his friends, are acute and discriminating. One of the most interesting events in the professional life of Dr. Combe was his appointment as physician to Leopold, King of Belgium, in the year 1835. His frail health prevented him from long enjoying this honor, and he was obliged in consequence to leave Brussels and return to Edinburgh. The short intercourse between the sovereign and his new physician was alike honorable to both. Encouragement for the exercise of a strong will, under the impelling motive of enlarged benevolence, is offered in the following passage by Sir James Clark: "Yet, by the judicious management of himself and frequent changes of climate, and by a careful adaptation of his mental and bodily labors to his powers of endurance during the periods of comparative health which he did possess, he continued, in a quiet, unostentatious way, to do much good during his too short life, and he has left behind him writings which are of the utmost value to mankind."

Dr. Combe paid a short visit to the United States in the early part of the summer of 1847. He passed a few days in Philadelphia; but such was the delicate state of his health that he was unable to meet his professional brethren of this city at even a small reunion projected for him by the writer of the present article.

